# Identification of a methyltransferase-related long noncoding RNA signature as a novel prognosis biomarker for lung adenocarcinoma

**DOI:** 10.18632/aging.205837

**Published:** 2024-05-20

**Authors:** Yang Yong Sun, Shuang Li, Chang Liu, Yaqiang Pan, Ying Xiao

**Affiliations:** 1 Department of Cardiothoracic Surgery, Affiliated People’s Hospital of Jiangsu University, Zhenjiang, China; 2 Department of Cardiology, First Affiliated Hospital of Xinjiang Medical University, Xinjiang, China; 3 Department of Emergency, Nanjing Jiangning Hospital, Jiangsu, China

**Keywords:** lung adenocarcinoma, methyltransferase-related lncRNAs, prognosis, GSEA, immune infiltration

## Abstract

Background: Lung adenocarcinoma (LUAD) accounts for a high proportion of tumor deaths globally, while methyltransferase-related lncRNAs in LUAD were poorly studied.

Methods: In our study, we focused on two distinct cohorts, TCGA-LUAD and GSE3021, to establish a signature of methyltransferase-related long non-coding RNAs (MeRlncRNAs) in LUAD. We employed univariate Cox and LASSO regression analyses as our main analytical tools. The GSE30219 cohort served as the validation cohort for our findings. Furthermore, to explore the differential pathway enrichments between groups stratified by risk, we utilized Gene Set Enrichment Analysis (GSEA). Additionally, single-sample GSEA (ssGSEA) was conducted to assess the immune infiltration landscape within each sample. Reverse transcription quantitative PCR (RT-qPCR) was also performed to verify the expression of prognostic lncRNAs in both clinically normal and LUAD samples.

Results: In LUAD, we identified a set of 32 MeRlncRNAs. We further narrowed our focus to six prognostic lncRNAs to develop gene signatures. The TCGA-LUAD cohort and GSE30219 were utilized to validate the risk score model derived from these signatures. Our analysis showed that the risk score served as an independent prognostic factor, linked to immune-related pathways. Additionally, the analysis of immune infiltration revealed that the immune landscape in high-risk groups was suppressed, which could contribute to poorer prognoses. We also constructed a regulatory network comprising 6 prognostic lncRNAs, 19 miRNAs, and 21 mRNAs. Confirmatory RT-qPCR results aligned with public database findings, verifying the expression of these prognostic lncRNAs in the samples.

Conclusion: The prognostic gene signature of LUAD associated with MeRlncRNAs that we provided, may offer us a comprehensive picture of the prognosis prediction for LUAD patients.

## INTRODUCTION

Over the epochs, lung cancer has predominantly reigned supreme in global incidence, unequivocally establishing itself as the paramount cause of tumor-induced fatalities [1]. Routinely, lung cancer can be classified as many pathological subtypes, such as small cell lung cancer (SCLC) and non-small cell lung cancer (NSCLC), among which the common histological subtype is lung adenocarcinoma (LUAD), with an incidence of about 50% of the total cases of lung cancer [2]. Although the diagnosis and treatment technology of LUAD is constantly improving compared with the past, the mortality rate has not decreased significantly [3]. Even though molecular targeted therapy has made tremendous advances as well as immunotherapy, the overall survival (OS) remains unsatisfactory on account of a defect of new biological indicators associated with the prognosis of LUAD. In addition, LUAD is nowadays diagnosed in the advanced stage without the opportunity for surgical treatment, while the original tumor focus has already been transmitted to nearby tissue or organ [4]. Therefore, it is crucial to identify the key prognostic indicators for LUAD.

Recent advancements in genomic and transcriptomic analyses have unveiled the complex landscape of long non-coding RNAs (lncRNAs) and their pivotal roles in various biological processes, including the progression and pathogenesis of LUAD [5, 6]. Additionally, lncRNAs have biological repertoires in malignant tumor immunology, including tumor antigen expression, immunological escape, immune checkpoint, and infiltration. As a result, they may have a great potential to be a biomarker to determine the prognosis [7]. Recently, it was shown that methyltransferase-relevant long noncoding RNA (MeRlncRNA) regulators harness their strengths to promote the occurrence and progress of glioma and are critical for determining prognosis and therapeutic approach [8]. A series of enzymes have been proven to target certain specific lncRNAs, such as methyltransferase-like 3 and DNA methyltransferase-like 2 [9]. Among these, MeRlncRNAs have emerged as critical players in the epigenetic regulation of gene expression, influencing tumorigenesis, metastasis, and response to therapy in LUAD [10, 11]. Moreover, the dysregulation of MeRlncRNAs has been correlated with patient prognosis, suggesting their potential as novel biomarkers for LUAD diagnosis and prognosis prediction. For instance, the expression of Methyltransferase-like 1 not only advanced in LUAD but also the degree of increase was inversely proportional to the prognosis of cancer patients [12]. Despite their significance, the roles of MeRlncRNAs in LUAD remain inadequately explored, necessitating further investigation to elucidate their mechanisms of action and their implications in lung adenocarcinoma pathophysiology. This study aims to bridge this gap by identifying and characterizing a signature of MeRlncRNAs associated with the prognosis of LUAD patients, thereby contributing to a more comprehensive understanding of their biological functions and clinical relevance.

Numerous details on tumors, including gene expression, methylation, mutation, and clinical characteristics, are available via The Cancer Genome Atlas (TCGA) and Gene Expression Omnibus (GEO). Our research identified MeRlncRNAs in LUAD for the first time and then developed a significant MeRlncRNAs-related genes prognostic model using univariate Cox and the least absolute shrinkage and selection operator (LASSO). We also have verified the availability of the model via internal and external cohorts. Besides, this study explored the association of risk score and clinical characteristics, further examination as a prognostic marker and independent of other clinical features, and successfully constructed a nomogram in LUAD. Interestingly, the results of GSEA employed to explore the mechanism of prognostic differences between high- and low-risk sets showed that the immune microenvironment of the high-risk group was inhibited, which probably was the cause of the dreadful prognosis. Finally, we performed an RT-qPCR trial to quantitatively detect the expression of six prognostic lncRNAs in LUAD tissues and control lung tissues and verified the consistency of the quantitative results with the gene database data. Intentionally, based on transcriptome data from public databases and corresponding clinical information, bioinformatics methods were used to establish methyltransferase-related lncRNA gene signatures for envisaging the prognosis for LUAD patients, thereby laying the groundwork for clinical prognosis and pinpointed therapeutic interventions.

## MATERIALS AND METHODS

### Data source

All available clinical information and public transcriptome data were derived from the GEO and TCGA databases, respectively. Two cohorts, namely, and TCGA-LUAD and GSE30219, were enrolled in this study. The TCGA-LUAD cohort contains 59 normal and 483 LUAD samples. The GSE30129 cohort including 293 LUAD samples with survival information was utilized as the validation cohort. Methyltransferase-related genes were acquired from MsigDB (http://www.broad.mit.edu/gsea/msigdb/) from the following 13 gene set entries: ‘go mRNA methyltransferase activity’, ‘go RNA 2o methyltransferase activity’, ‘go RNA methyltransferase activity’, ‘go rRNA adenine methyltransferase activity’, ‘go rRNA cytosine methyltransferase activity’, ‘go rRNA guanine methyltransferase activity’, ‘go tRNA adenine methyltransferase activity’, ‘go tRNA cytosine methyltransferase activity’, ‘go tRNA guanine methyltransferase activity’, ‘go tRNA methyltransferase activity’, ‘go tRNA methyltransferase complex’, ‘gocc methylosome’, and ‘gocc methyltransferase complex’.

### Identification of differentially expressed genes (DEGs), lncRNA (DELncRNAs), and methyltransferase-related lncRNAs (MeRlncRNAs) in LUAD

Screening the DEGs and DELncRNAs was executed in the ‘limma’ package [13, 14]. The threshold for DEGs was adjusted *p* < 0.05 and |Log_2_FC| >1. The volcano map was created by the ‘ggplot2’ package. The ‘heatmap’ package was utilized to plot the heatmap of top 50 up-regulated and top 50 down-regulated genes. The identification of differentially expressed methyltransferase-related genes was achieved through the intersection of the differentially expressed genes (DEGs) and methyltransferase-related genes. The Pearson correlation between differentially expressed methyltransferase-related genes and DElncRNAs was calculated, and the relationship pairs with |Correlation coefficient| >0.3 and *p* < 0.05 were screened to build the mRNA-lncRNA network and obtain MeRlncRNAs.

### Construction and verification of the gene signature based on MeRlncRNAs

A total of 483 patients diagnosed with LUAD were subjected to random allocation, with 145 patients assigned to the test set and 338 patients assigned to the training set, maintaining a proportion of 7:3. For the identification of prognostic MeRlncRNAs, we implemented a two-step approach: initially, univariate Cox regression analysis was conducted to assess the association between the expression levels of each MeRlncRNA and overall survival in LUAD patients. MeRlncRNAs with a *P*-value < 0.05 in this analysis were deemed potentially prognostic and subjected to further evaluation using LASSO regression analysis. LASSO regression using the ‘glmnet’ R package, known for its efficacy in handling high-dimensional data, was applied to refine the selection of MeRlncRNAs by penalizing the regression coefficients, thus preventing overfitting and enhancing the model’s predictive accuracy. Riskscore = β_1_X_1_ + β_2_X_2_ + … + β_n_X_n_. was the formula for computing risk scores. β denotes the regression coefficient, and X_1_ represents the expression level of prognosis-related lncRNA.

In our study, patient stratification was based on the median value of the risk score, which served as a critical metric for dividing patients into low-risk and high-risk groups. To visualize and analyze the survival differences between these groups, Kaplan-Meier (K-M) survival curves were generated, and the statistical significance of differences in survival was assessed using the log-rank test. Additionally, the ‘survivalROC’ package in R was employed to calculate the area under the curve (AUC), providing a quantitative measure of the prognostic signature’s accuracy in predicting patient outcomes. To further illustrate the distribution of risk scores and their correlation with patient outcomes, risk plots were created utilizing the ‘heatmap’ package in R. These plots offered a visual representation of the risk score distribution across patients, alongside key clinical features, thus facilitating a comprehensive analysis of the prognostic model’s performance.

For external validation of our prognostic model, the GSE30219 cohort was utilized as the validation cohort. In the GEO cohort, participants were divided into low-risk and high-risk groups, using the median as the dividing criterion. This step was crucial for assessing the model’s generalizability and reliability across different patient populations, ensuring that our findings hold potential clinical relevance beyond the initial study cohort.

### Risk score and clinicopathological parameters correlation

The association between risk scores and clinical features, including gender, age, pathological stage, and TNM classification, was determined using the Wilcoxon test.

### Prognostic analysis and nomogram construction

Utilizing the ‘survminer’ package in R, both univariate and multivariate Cox regression analyses were conducted to identify independent predictors of OS. Subsequently, a nomogram incorporating these independent prognostic factors was developed with the ‘rms’ package in R.

### Gene set enrichment analysis (GSEA)

The ‘GSEA’ package was employed to identify significantly enriched pathways in LUAD samples compared to high- and low-risk samples based on the expression differences of MeRlncRNAs. We used the Molecular Signatures Database (MSigDB) curated gene sets collection (KEGG and Hallmark gene sets) as the reference for pathway analysis. The analysis parameters were set to 1000 permutations to estimate the enrichment score (ES) significance, with a nominal *p*-value < 0.05 |NES| >1 considered statistically significant.

### Estimation of immune cell infiltration

For the assessment of immune infiltration landscapes, we applied ssGSEA using the ‘GSVA’ package in R. This method allows the estimation of pathway activity levels in individual samples based on their gene expression profiles. We used a predefined gene set comprising genes associated with immune cell types and functions. The ssGSEA scores were calculated for each sample to derive the immune infiltration landscape, facilitating the comparison between high-risk and low-risk groups as defined by the prognostic gene signature. Depictions of varying immune cell penetrations emerged through box plots. Risk score and immune cells were calculated to have a Pearson association.

### Construction of the lncRNA-miRNA-mRNA regulatory network

We first used Miranda to predict the miRNAs targeted by the prognostic lncRNAs and then used Starbase to predict the mRNAs targeted by the miRNAs. Using Cytoscape software, combined with methyltransferase-related genes, a lncRNA-miRNA-mRNA regulatory network was created.

### RNA preparation and quantitative real-time polymerase chain reaction (RT-qPCR)

ServiceBio Inc.’s nuclezol ls RNA isolation reagent was used to isolate total RNA from the 20 samples, including 10 normal and 10 LUAD tissue. The SureScript-First-strand-cDNA-synthesis-kit (ServiceBio Inc.) was then used to reverse transcribe total RNA into cDNA. After that, qPCR was carried out using GeneCopoeia’s BlazeTaqTM SYBR^®^ Green qPCR Mix 2.0. A succession of procedures, from RNA reverse transcription to thermocycling, was meticulously undertaken. Primer sequences were elucidated in Table 1. The relative expression level was evaluated by comparative 2^−ΔΔCT^ approach [15].

**Table 1:** The primer sequences for 6 lncRNAs and GAPDH.

**Symbol**	**Forward**	**Reverse**
RP11-251M1.1	CCTGTGCTTTTTCACCTCTACG	GCCATTTTTTCCATTTTTTTCC
RP1-78014.1	CAGAGAGAAGGATGGAGTGGG	GTTTATTTTGCTGTGCAGAAA
LINC00511	GGGTAGTAGGAGTGGGGTGG	CGCAGGAGATGTGATTGAGC
CTD-2510F5.4	TCACAGTGACCTGCTATGGACT	CAACATGAACCTTATATTTTCG
LINC01936	AGGAAGGGAGGAAAACAATA	AACTCAACATCCGACGAAAA
RP11-750H9.5	CAGAATGAAATGGAGCCACA	GGGAGAAACAGGACAAGAGG
GAPDH	CCCATCACCATCTTCCAGG	CATCACGCCACAGTTTCCC

### Statistical analysis

All analyses were conducted using R software. The Wilcoxon test was applied for comparing data between groups. A *p*-value < 0.05 was considered statistically significant, except in cases where specific circumstances dictate otherwise.

### Data availability statement

All data generated or analysed during this study are available in the TCGA.

## RESULTS

### Identification of methyltransferase-related lncRNAs (MeRlncRNAs) in LUAD

**Figure 1:**
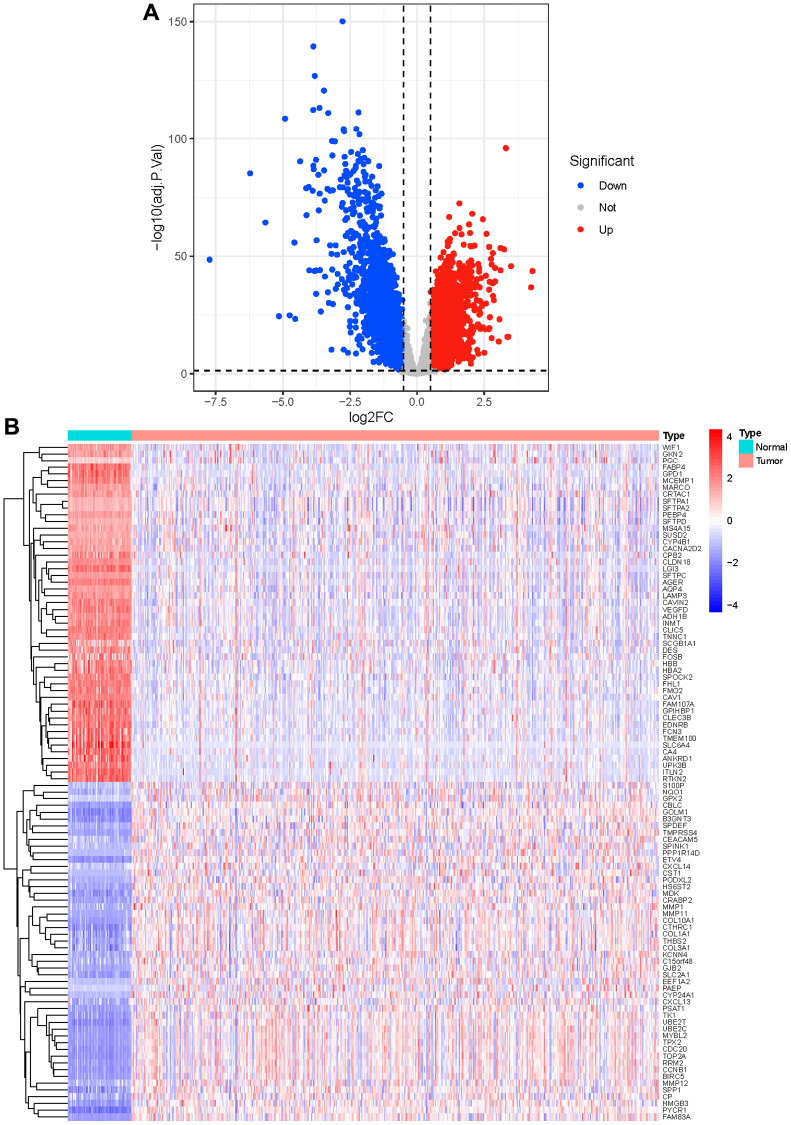
**Identification of differential genes.** (**A**) The red dots in the plot represent up-regulated genes and blue dots represent down-regulated genes with statistical significance. Gray dots represent no DEGs; (**B**) The heatmap of top 50 up-regulated and top 50 down-regulated genes in tumor and normal tissue.

**Figure 2:**
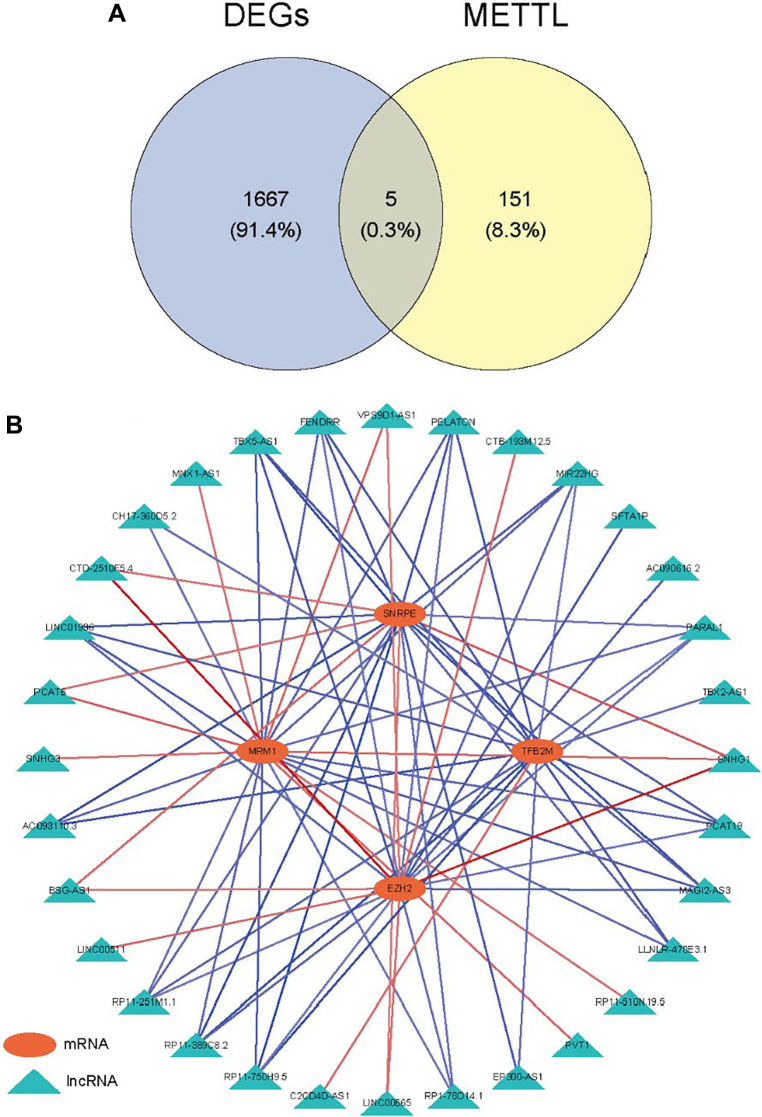
**Identification of methyltransferase-related lncRNAs.** (**A**) Venn diagram of the intersection between DEGs and methyltransferase-related genes; (**B**) The mRNA-lncRNA network.

Compared with the normal samples, 741 up-genes and 931 under-repressed genes were mined from the LUAD samples, with a total of 1672 DEGs (Supplementary Table 1 and Figure 1A). The top 50 genes each with the most significant differences in up-regulated or down-regulated expression were taken to draw a heat map, as shown in Figure 1B. Then, we obtained 156 methyltransferase-related genes from MsigDB after de-duplication (Supplementary Table 2). Hence, five differentially expressed methyltransferase-related genes (SNRPE, TFB2M, EZH2, MRM1, and METTL1) were discovered by intersecting the 1672 DEGs and 156 methyltransferase-related genes (Figure 2A). Meanwhile, 87 DElncRNAs between normal and LUAD samples were also extracted and listed in Supplementary Table 3. In the following, the Pearson correlation between the above five methyltransferase-related genes and DElncRNAs was calculated. To build the mRNA-lncRNA network, relationship pairs with |Correlation coefficient| >0.3 and *p* < 0.05 were chosen. (Supplementary Table 4). Finally, an mRNA-lncRNA network containing 36 nodes (32 lncRNAs and 4 mRNAs) and 80 edges was generated (Figure 2B). The 32 lncRNAs in the network were defined as methyltransferase-related lncRNAs (MeRlncRNAs) in LUAD for subsequent analysis.

### Construction of prognostic signature based on MeRlncRNAs

**Figure 3:**
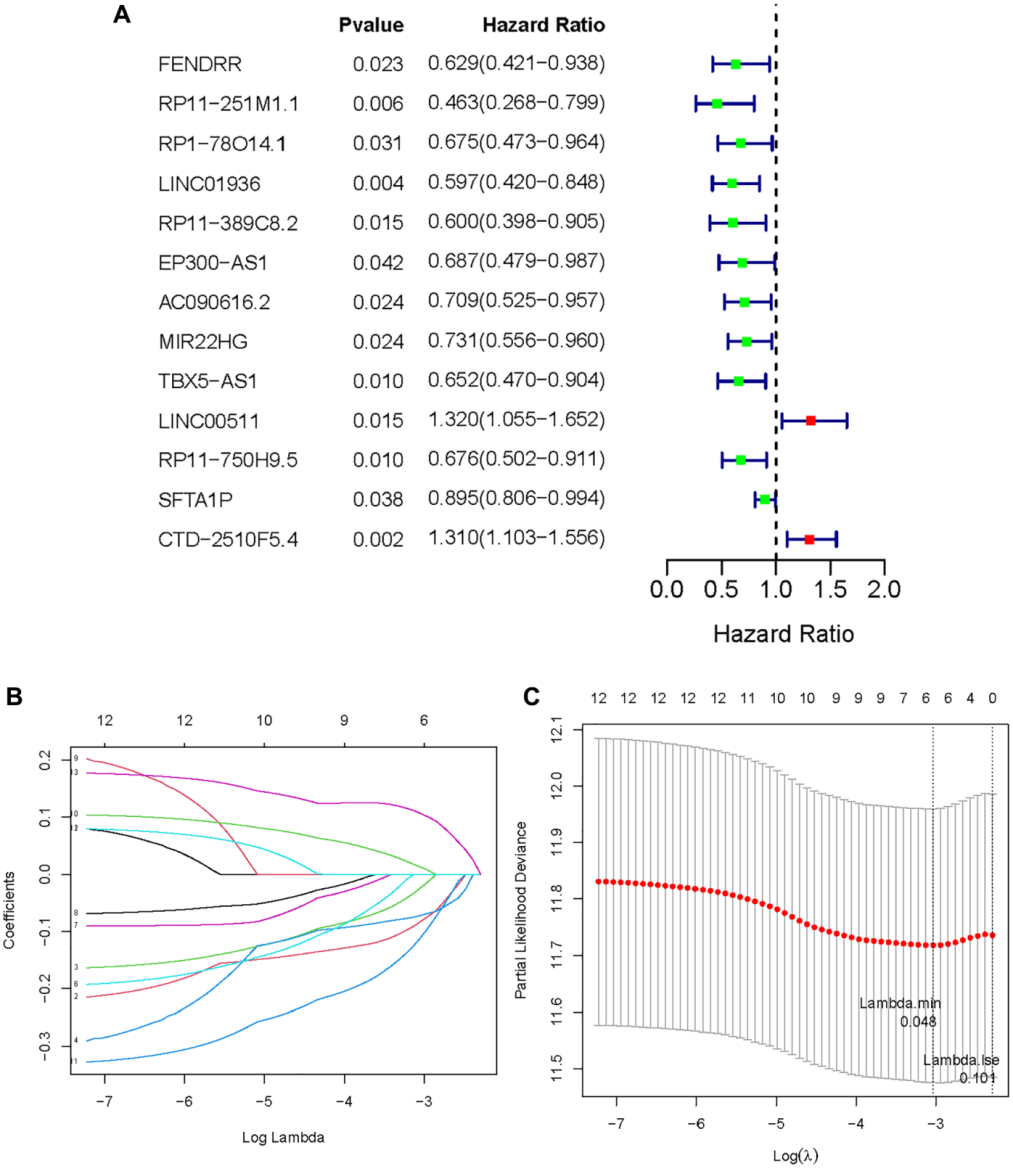
**Establishment of methyltransferase-related lncRNAs signature.** (**A**) A forest plot of prognostic methyltransferase-related lncRNAs identified by univariate Cox and Kaplan-Meier survival analysis; (**B**, **C**) LASSO regression analysis.

A training set of 338 specimens and a testing set of 145 individuals were created from the 483 LUAD patients in the TCGA cohort. After that, 32 MeRlncRNAs from the training cohort were used in the univariate Cox regression analysis to find out the lncRNAs associated with prognosis. In the training set, the OS of cancer patients was shown to be strongly correlated with 13 out of the 32 MeRlncRNAs. (Figure 3A). Then, the 13 lncRNAs were further submitted to LASSO regression analysis. Six MeRlncRNAs (RP11-251M1.1, RP1-78014.1, LINC01936, LINC00511, RP11-750H9.5, and CTD-2510F5.4) were identified as prognostic lncRNAs (Figure 3B) with lambda.min was 0.048 (Figure 3C). Details such as regression coefficient can be found in the Supplementary Table (Supplementary Table 5). The model was constructed by the formula: Riskscore = −0.081695395 × RP11-251M1.1 + −0.022561732 × RP1-78014.1 + −0.071522442 × LINC01936 + 0.012186292 × LINC00511 + −0.10909988 × RP11-750H9.5 + 0.105977347 × CTD-2510F5.4.

**Figure 4:**
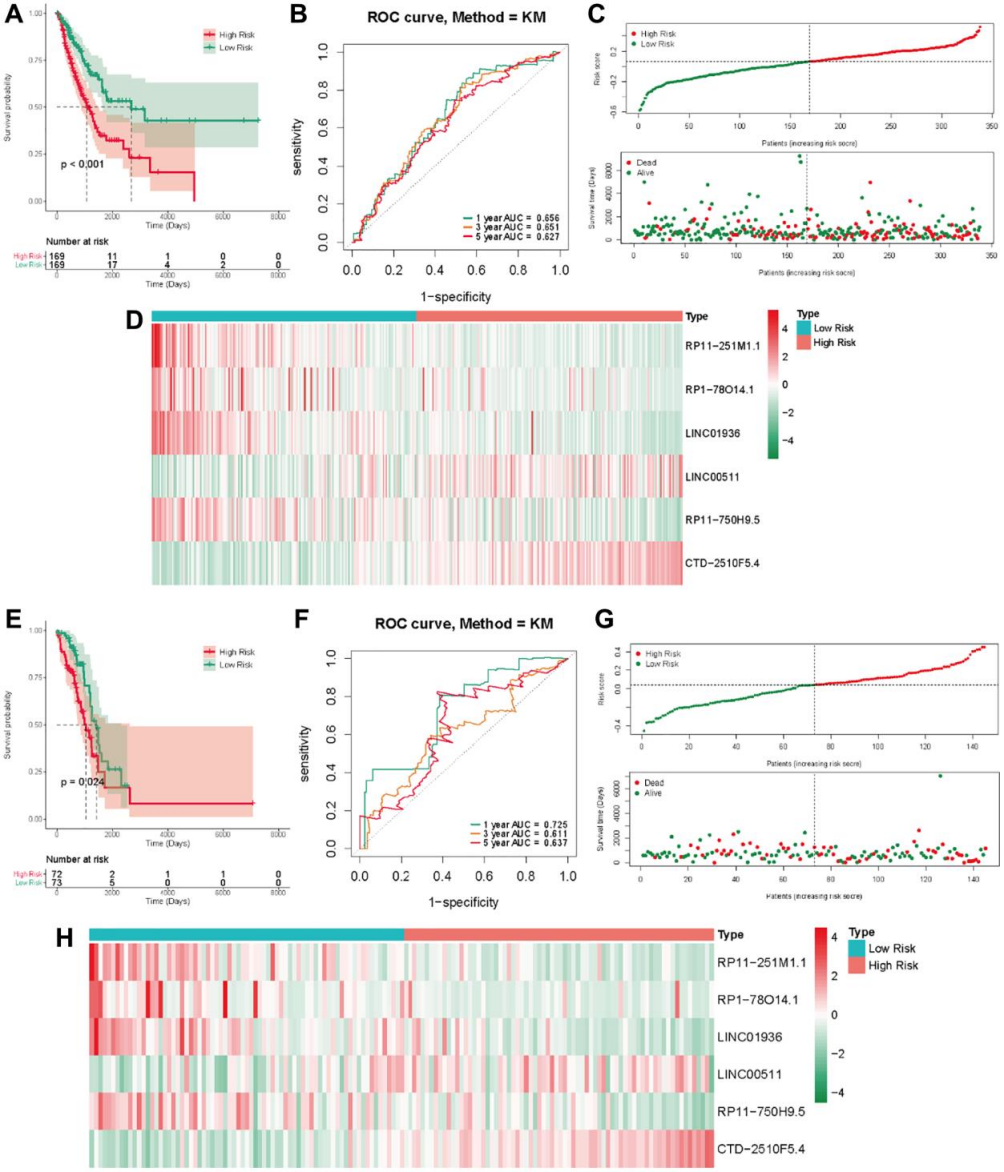
**The methyltransferase-related IncRNAs signature was a prognostic biomarker for OS in the TCGA-LUAD cohort.** (**A**) K-M survival of OS according to methyltransferase-related IncRNA signature groups in the training cohorts; (**B**) AUC of time-dependent ROC curve for the risk score in the training dataset; (**C**) The OS status and OS risk score plots in the training dataset; (**D**) The heat map of these 6 methyltransferase-related lncRNAs between the high- and low-risk groups in the training dataset; (**E**) K-M survival of OS according to methyltransferase-related IncRNA signature groups in the test cohorts; (**F**) AUC of time-dependent ROC curve for the risk score in the test dataset; (**G**) The OS status and OS risk score plots in the test dataset; (**H**) The heat map of these 6 methyltransferase-related lncRNAs between the high- and low-risk groups in the test dataset.

In the TCGA-LUAD cohort, the training group was divided into a low-risk group and a high-risk group, using the median as the dividing criterion. High-risk patients have worse prognosis than low-risk patients (Figure 4A). In the training set, the AUC values for 1, 3 and 5 years were 0.656, 0.651, and 0.627, respectively (Figure 4B). Figure 4C visually demonstrates the risk score and survival status in the training cohorts. The survival status distribution plot shows that as the risk score increases, patients face a greater risk of death. Figure 4D shows the expression of six prognostic lncRNAs. Transcription analysis results showed that the expression of LINC00511 and CTD-2510F5.4 was up-regulated in the high-risk group, while the expression of RP11-251M1.1, RP1-78014.1, LINC01936 and RP11 750H9.5 was up-regulated in the low-risk group. At the same time, the test group also showed similar results to the training group (Figure 4E–4H).

### External validation in the GSE13507 cohort

**Figure 5:**
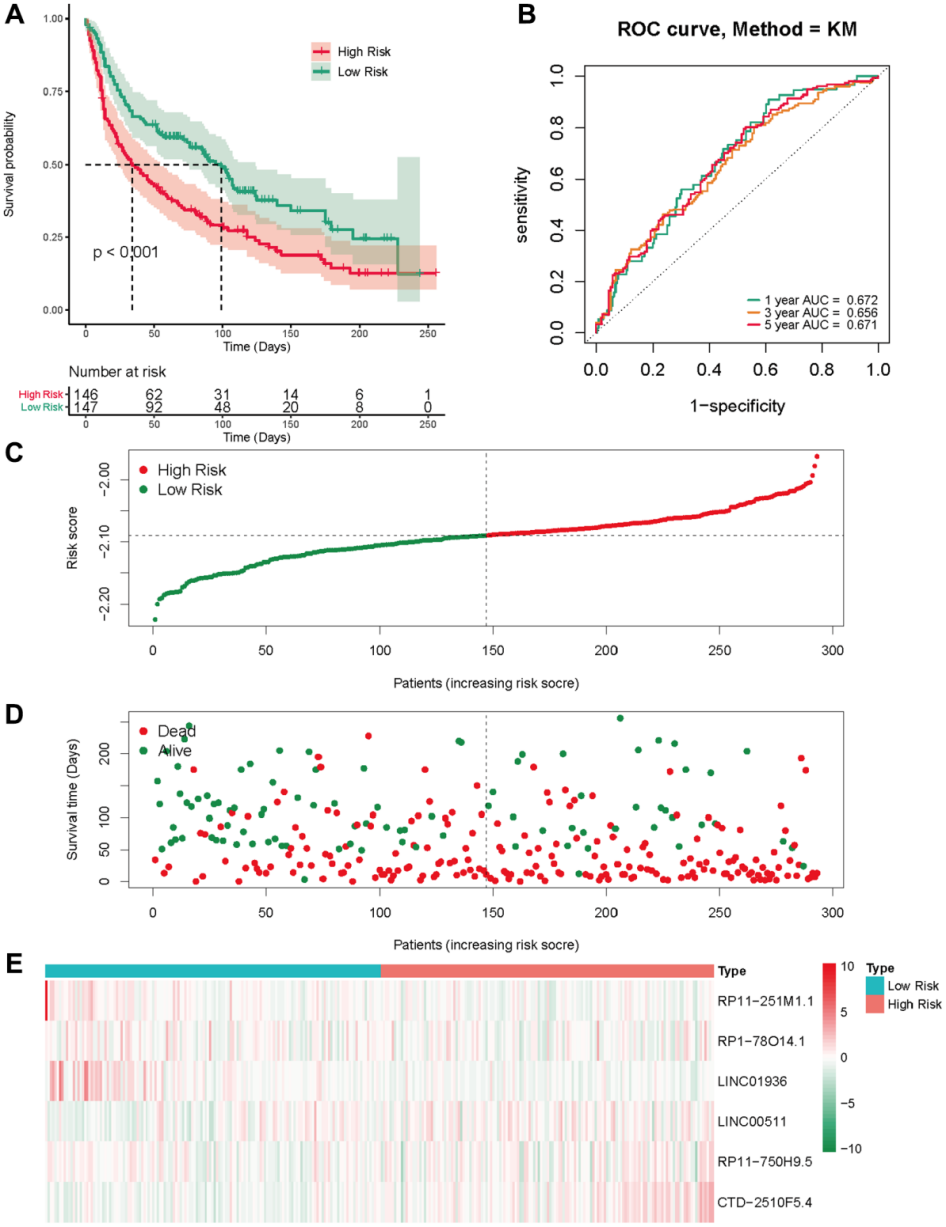
**External validation of the risk score in the GSE30219 cohort.** (**A**) The Kaplan-Meier survival analysis; (**B**) The time-dependent ROC analysis for the risk score in predicting the OS of patients in the GSE30219 cohort; (**C**, **D**) The risk score distribution and survival status of patients in the GSE30219 cohort; (**E**) The heatmap analysis.

We employed a separate cohort made up of 293 lung cancer patients from the GSE13507 to confirm the model’s external applicability. Those with a heightened risk metric evidenced a compromised OS, echoing findings from TCGA cohorts (Figure 5A). Accordingly, the AUC estimations, for one, three, and five years, were recorded as 0.672, 0.656, and 0.671, respectively (Figure 5B). Figure 5C, 5D shows that the number of deaths increases as the risk score increases in the external data validation. Figure 5E shows the difference in expression levels of six prognosis-related lncRNAs in high and low risk groups. These outcomes further supported the ability and reliability of MeRlncRNAs-related risk models to predict 1-year, 3-year, and 5-year survival of patients.

### Relationship between the gene signature and clinical characteristics

**Figure 6:**
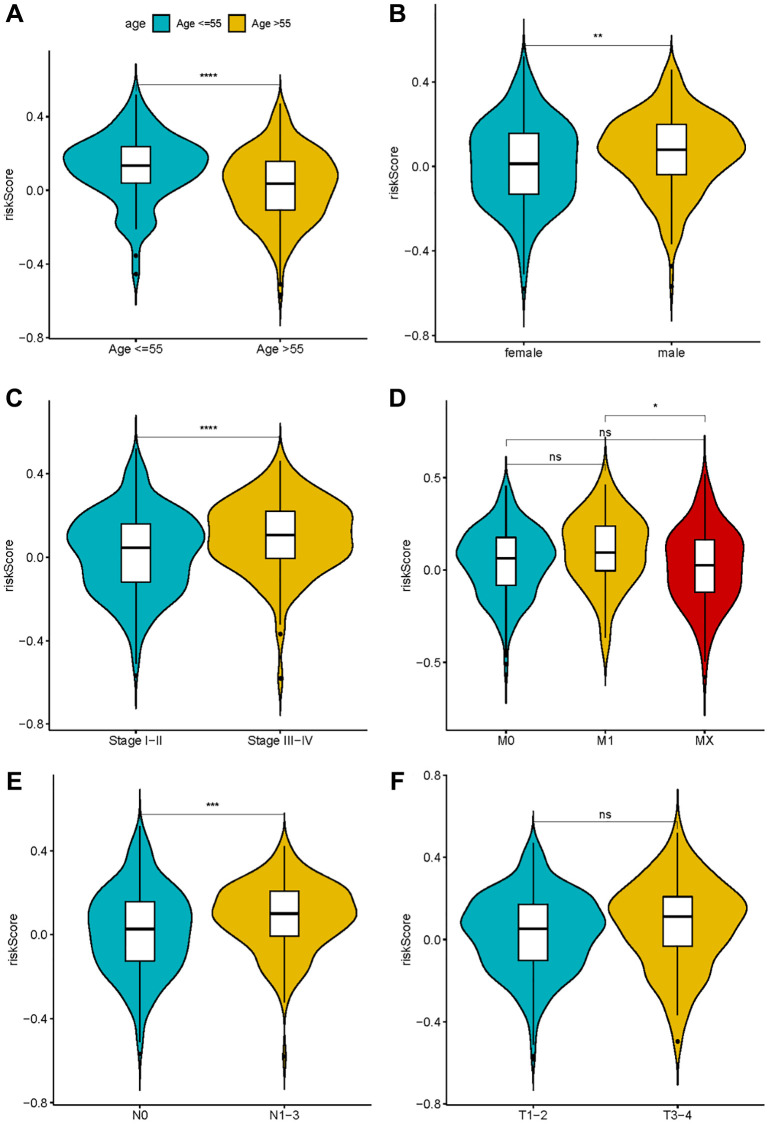
**The correlation between risk score and clinical characteristics.** (**A**) Age; (**B**) Sex; (**C**–**F**) TNM stage.

Exploring the association between risk score and clinical characteristics can better comprehend the significance of gene signature in the occurrence and development of LUAD. As shown in Figure 6, the risk score was augmented amongst males, individuals surpassing 55 years, and those grappling with high N stage, consolidating the conclusion that the risk score is associated with factors such as gender, age, and tumor malignancy.

### Independent prognosis and nomogram construction

**Figure 7:**
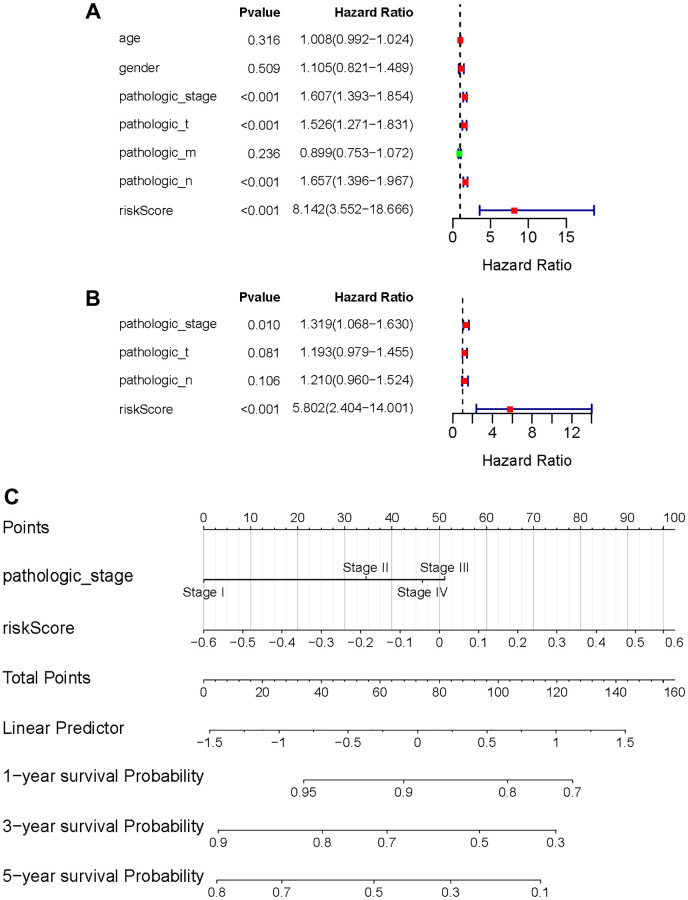
**Independent value of the prognostic risk model.** (**A**, **B**) Forrest plots of the univariate Cox regression analysis; (**B**) Forrest plot of the multivariate Cox regression analysis; (**C**) The nomogram was established based on the independent prognosis model.

Following that, univariate and multivariate Cox regression analysis was used to determine whether the risk score was a prognostic factor for LUAD patients when independent of other clinical factors in this study. Pathological stage, T phase, N phase, and the risk metric were all intertwined with patient fate (Figure 7A). Multivariate Cox regression analysis results emphasize the possibility that risk score can independently predict the prognosis of patients (Figure 7B). Furthermore, in the multivariate analysis, the pathologic stage was determined to be a significant prognostic predictor. Then, using independent prognostic markers such as pathologic stage and risk score, we built a nomogram that could predict patients OS in one, three, and five years (Figure 7C). The value obtained by calculating the C index of the nomogram was 0.6966719, suggesting an excellent accuracy in predicting patient survival.

### GSEA calculation results of high- and low-risk group

**Figure 8:**
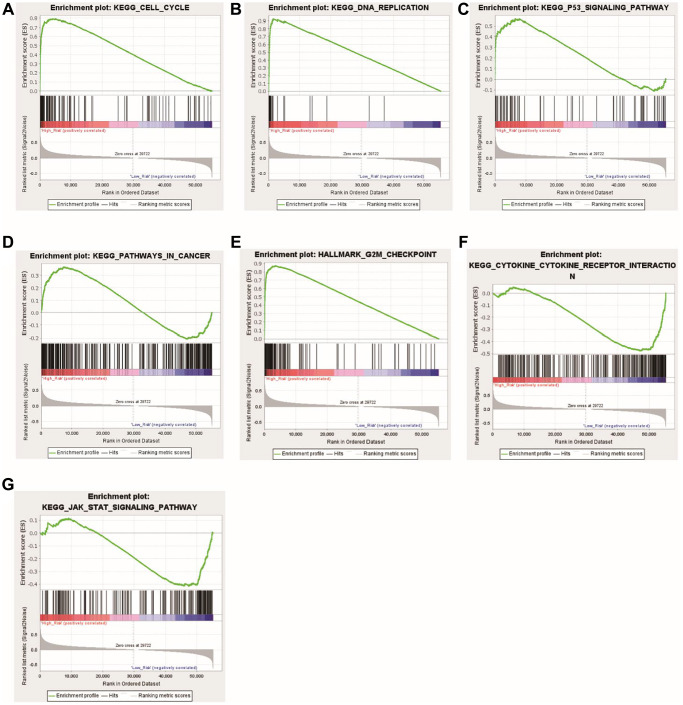
**Functional enrichment analyses between the high- and low-risk groups.** (**A**) KEGG_CELL_CYCLE; (**B**) KEGG_DNA_REPLICATIO; (**C**) KEGG_P53_SIGNALING_PATHWAY; (**D**) KEGG_PATHWAYS_IN_CANCER; (**E**) HALLMARK_G2M_CHECKPOINT; (**F**) KEGG_CYTOKINE_CYTOKINE_RECEPTOR_INTERACTION; (**G**) KEGG_JAK_STAT_SIGNALING_PATHWAY.

The enrichment pathways of the high- and low-risk categories are listed in Supplementary Table 6. Among them, the high-risk consortium manifested enrichment in terminologies like ‘kegg_cell_cycle’, ‘kegg_dna_replication’, ‘kegg_p53_signaling_pathway’, ‘kegg_pathways_in_cancer’, and ‘hallmark_g2m_ checkpoint’ (Figure 8A–8E). The Opposite group showcased elevated indices of immune-centric pathways like “kegg_cytokine_cytokine_receptor_interaction” and “kegg_jak_stat_signaling_pathway” (Figure 8F, 8G).

### The landscape of immune cell infiltration between the high- and low-risk group

**Figure 9:**
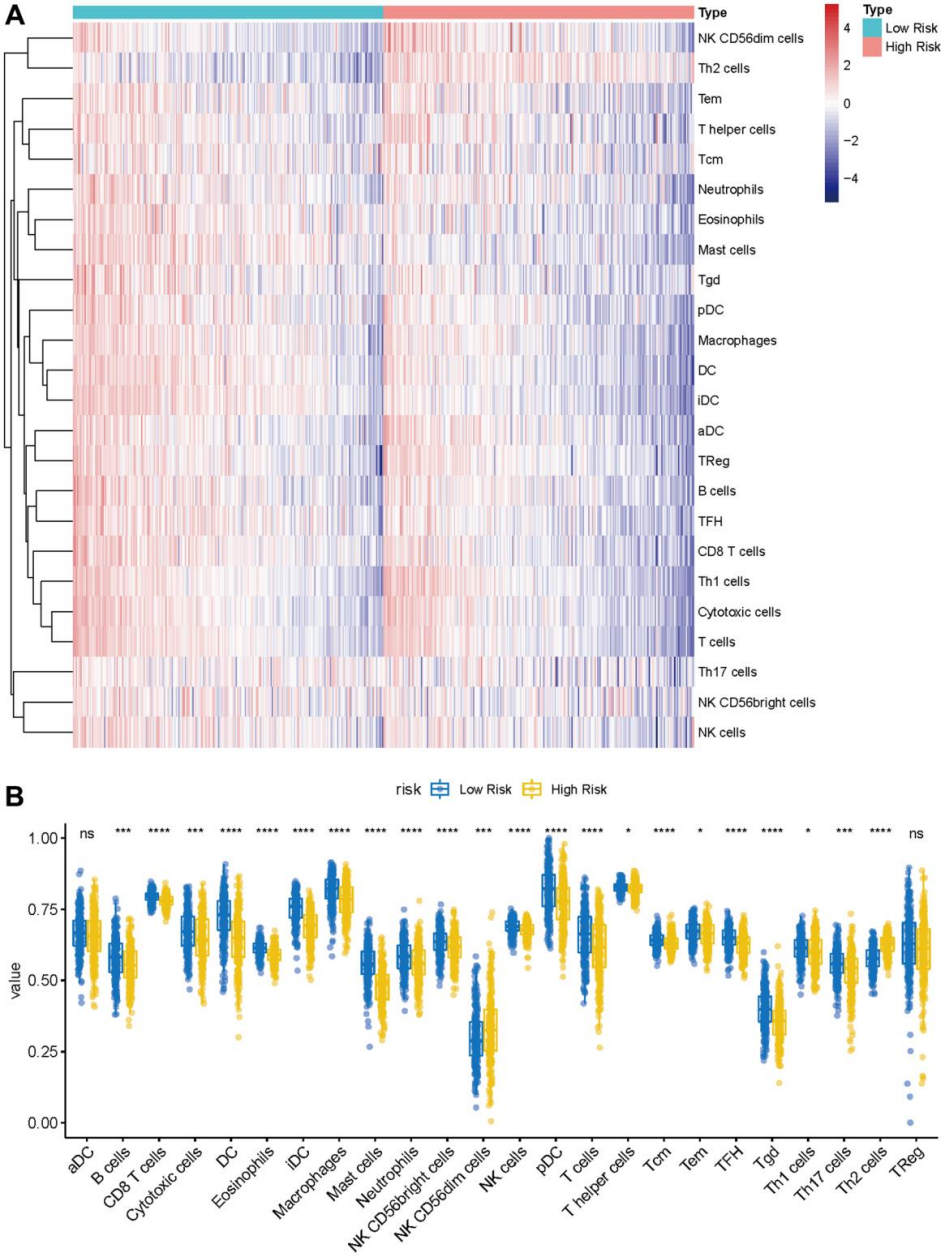
**Immune characteristics of methyltransferase-related lncRNAs-based classifier subgroups.** (**A**) The heatmap of immune infiltrating cells between the high- and low-risk groups; (**B**) The proportions of 24 infiltrated immune cells and infiltration score in the high-and low-risk groups. ^*^P < 0.05; ^***^P < 0.001; ^****^P < 0.0001.

**Figure 10:**
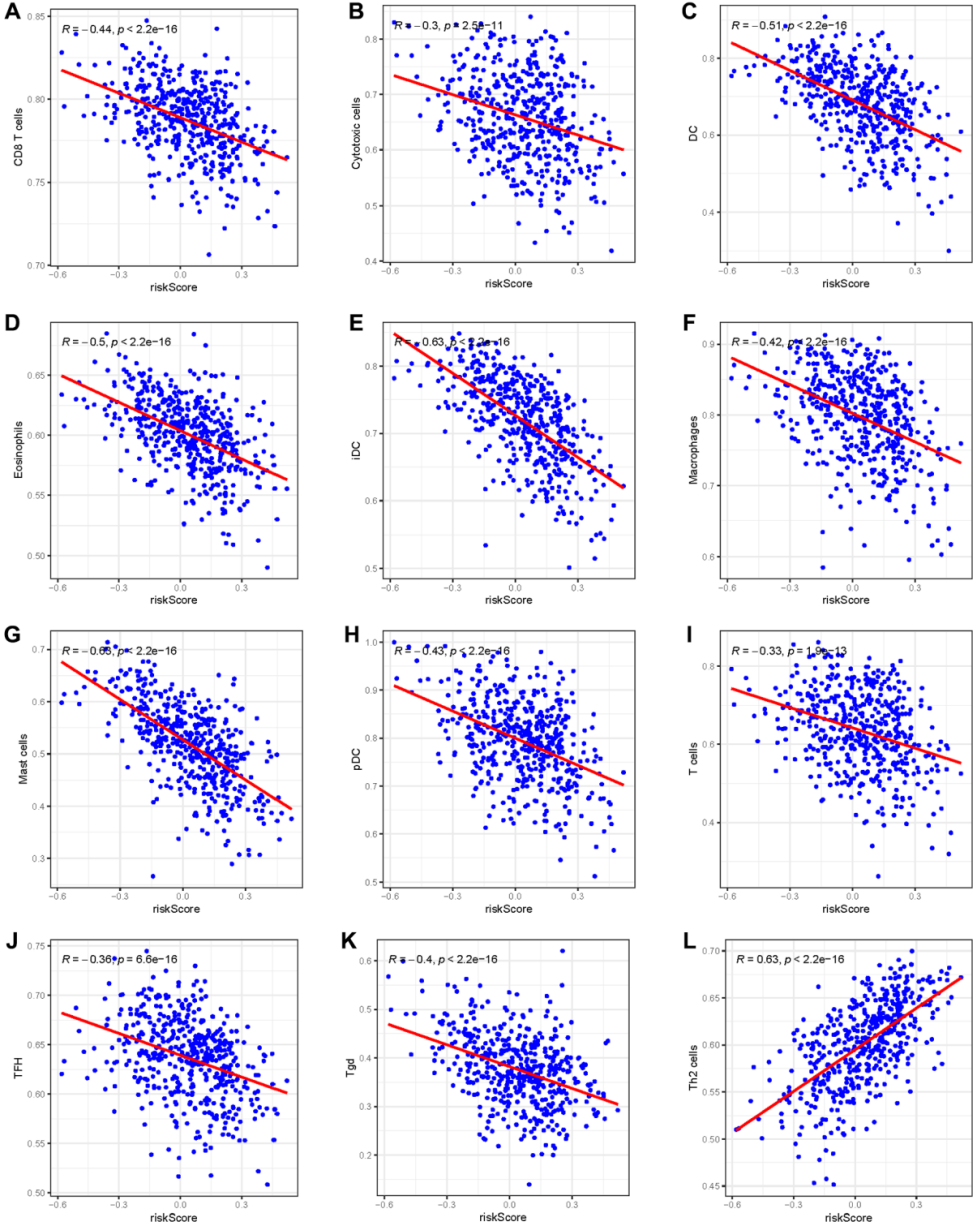
**The Pearson correlation between the risk score and immune cells.** (**A**) CD8 T cells; (**B**) Cytotoxic cells; (**C**) DC; (**D**) Eosinophils; (**E**) iDC; (**F**) Macrophages; (**G**) Mast cells; (**H**) pDC; (**I**) T cells; (**J**) TFH; (**K**) Tgd; (**L**) Th2 cells.

Given the prominence of immune pathways in GSEA, a differential examination of immune cell infiltration across risk factions was inaugurated. Findings intimated that the relative score of NK CD56dim cells and Th2 cells increased in the high-risk group, whereas metrics of an array of cells like B cells, Eosinophils, CD8 T cells, and Neutrophils dominated in the low-risk group (Figure 9A, 9B). These results suggest that the immunological milieu of individuals in the high-risk group is inhibited, which may be a factor in the prognosis’s bad outcome. A Pearson correlation was also deduced between the risk metric and various immune-related cells. The ensuing data indicated an inverse correlation between risk score and cells like CD8 T cells, Cytotoxic cells, DC, Eosinophils, iDC, Macrophages, Mast cells, pDC, T cells, TFH, and Tgd (Figure 10A–10K). Meanwhile, Th2 cells exhibited a symbiotic relationship with the risk score (Figure 10L).

### Construct the lncRNA-miRNA-mRNA regulatory network

**Figure 11:**
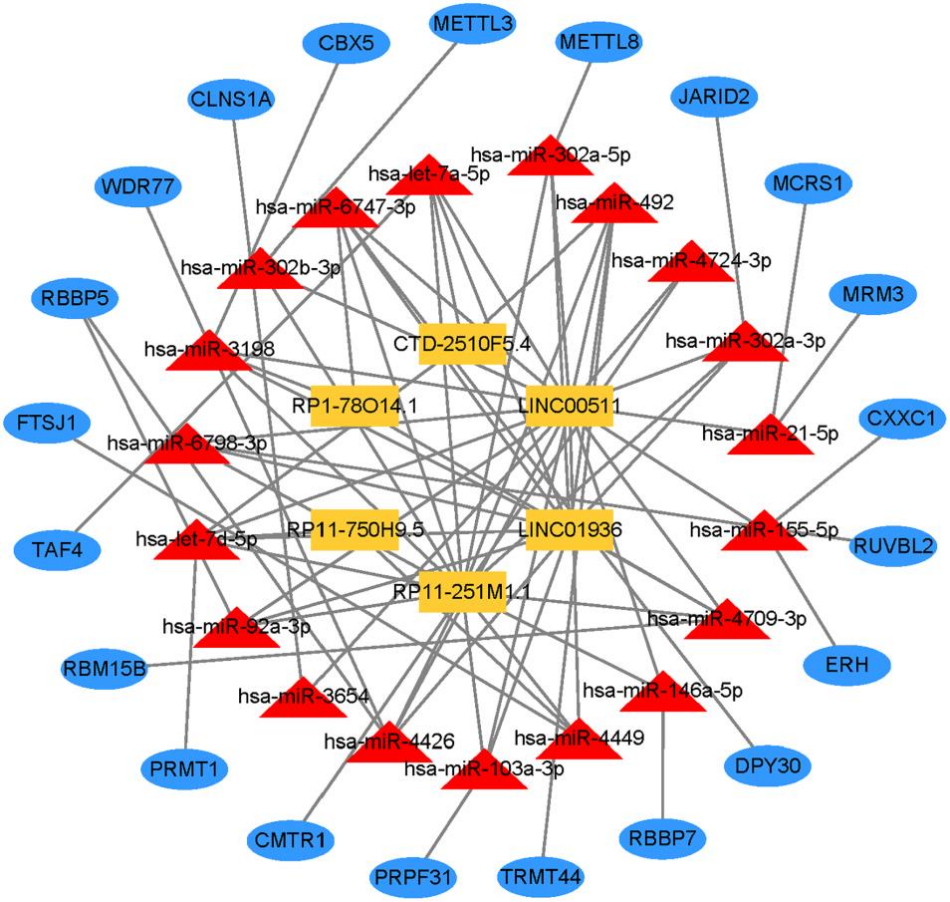
Prognostic lncRNA-miRNA-mRNA regulatory network in GC.

To further explore the ceRNA regulation mechanism based on six prognostic lncRNAs, we attempted to construct a lncRNA-miRNA-mRNA regulatory network. Combining the prediction result of the public database with lncRNAs related-methyltransferase, we finally created a regulatory network containing 6 prognostic lncRNAs, 19 miRNAs, and 21 mRNAs (Figure 11, Supplementary Table 7).

### Validation of the expression of lncRNAs

**Figure 12:**
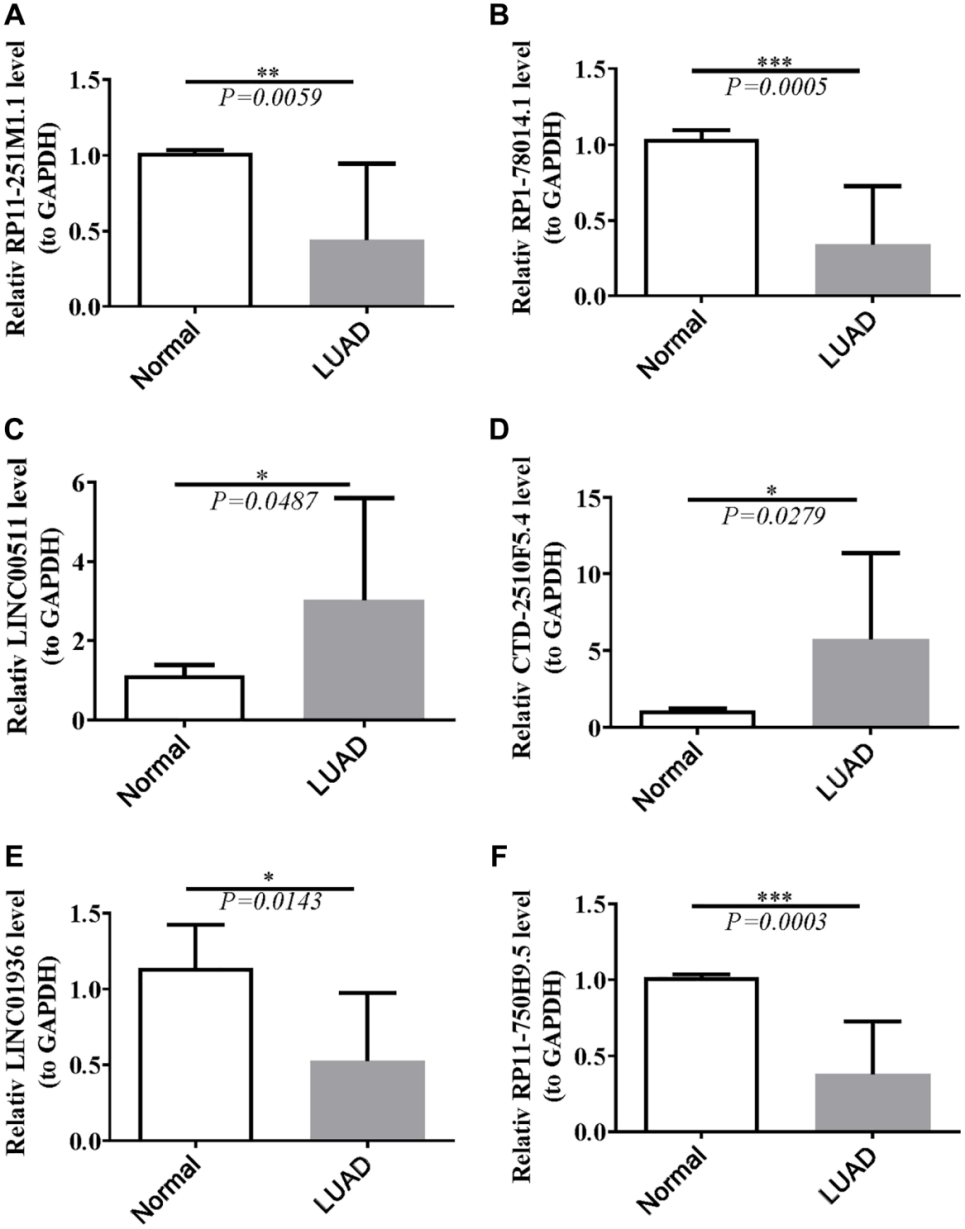
**Validation of lncRNAs’s expression.** (**A**) Statistical analysis of relative RP11-251M1.1 levels in HCC tissues compared to normal tissue controls; (**B**) The expression of RP1-78014.1 levels; (**C**) The expression of LINC00511 levels; (**D**) The expression of CTD-2510F5.4 levels; (**E**) The expression of LINC01936 levels; (**F**) The expression of RP11-750H9.5 levels. ^*^P < 0.05; ^**^P < 0.01; ^***^P < 0.001.

Data from the TCGA database showed that LINC00511 and CTD-2510F5.4 were up-regulated, as seen in Supplementary Table 3. Down-regulated in LUAD samples were RP11-251M1.1, RP1-78014.1, LINC01936, and RP11-750H9.5. Ten normal and ten LUAD samples were gathered, the RNA was extracted, and RT-qPCR was carried out aand the results further confirmed the findings. RP11-251M1.1, RP1-78014.1, LINC01936, and RP11-750H9.5 were down-regulated, as can be shown in Figure 12. When compared to normal samples, LINC00511 and CTD-2510F5.4 were more highly expressed in LUAD samples. In conclusion, the RT-qPCR results were in line with the information in the public database.

## DISCUSSION

The morbidity of LUAD, defined as the most elementary subtype of lung cancer, has gradually increased in recent decades. Despite a vast majority of therapeutic strategies, such as surgical operation, radiotherapy, chemotherapy, and targeted therapy, having been applied alternatively to LUAD patients, the OS has not been up to acceptable standards. Genetic alteration and immunological dysregulation in the humoral internal milieu are strongly associated with the development, invasion, and recurrence of LUAD [16]. Due to the crucial influence of humanity’s immune system on carcinoma advance [17], an array of immunotherapeutic therapies was executed to eliminate tumor cells to treat cancer [17]. However, attributed to the heterogeneity of biological characteristics of patients with LUAD [18], different individual has a different response to actual clinical immunotherapy, which means some patients might have an unfavorable therapeutic effect. In this pathway study, the mRNA-lncRNA network was constructed to preliminarily identify MeRlncRNA. Then a gene signature is closely related to immune-cell infiltration. Finally, we successfully performed RT-qPCR to verify the expression of prognostic MeRlncRNAs, which highly conformed to outcomes from the opening tumor database.

The burgeoning field of lncRNAs has garnered significant attention for their pivotal roles in tumorigenesis, tumor progression, and metastasis, positioning them as promising therapeutic targets and prognostic biomarkers for various malignancies [19]. Notably, aberrant expression of lncRNAs has been intricately linked to the immunopathologic dynamics of LUAD, suggesting their critical involvement in the disease’s immune microenvironment [20]. In a landmark study, Qian et al. [11] illuminated the landscape of lncRNA involvement in LUAD by identifying and characterizing LCAT3, a novel lncRNA significantly upregulated in LUAD tissues compared to adjacent normal tissues, and associated with a poor prognosis in lung cancer patients. LCAT3’s oncogenic potential was evidenced by its capacity to boost lung cancer cell proliferation, survival, migration, and invasion, effects that were mitigated by LCAT3 knockdown, leading to reduced tumor growth and metastasis in xenograft models.

Epigenetic modifications, involved in regulating gene extensive expression under the transcriptional level [21], consist of RNA methylation, gene silencing, genomic imprinting, and lncRNAs activities, and are thereby participating in tumorigenesis, progress, and metastasis [21]. Further investigation revealed that METTL3, a central player in the m6A methyltransferase complex, is upregulated in lung cancer and facilitates the m6A modification of LCAT3 [11]. This modification stabilizes LCAT3, elucidating a potential mechanism behind its overexpression in LUAD. The mechanistic pathways of LCAT3 extend to its direct interaction with FUBP1, which in turn upregulates c-MYC expression, a cornerstone oncogenic transcription factor implicated in cell proliferation, differentiation, and metabolism. The silencing of LCAT3 or FUBP1 markedly diminishes c-MYC levels, underscoring the critical LCAT3/FUBP1/c-MYC axis in lung cancer progression. Given the paramount importance of c-MYC in cellular regulatory mechanisms, targeting the LCAT3/FUBP1/c-MYC axis emerges as a novel and promising therapeutic strategy for LUAD. This research not only accentuates the critical role of lncRNAs and m6A modification in the oncological narrative but also charts a course towards the development of targeted LUAD therapies by disrupting the LCAT3/FUBP1/c-MYC network. Moreover, it casts a spotlight on the largely unexplored terrain of MeRlncRNAs and their potential interplay with immune regulation in LUAD’s immune microenvironment [21], paving the way for future investigations that could further unravel the complex molecular interactions at play in lung cancer.

As for exploring the functions of MeRlncRNAs mediation in the immune system about LUAD prognosis, we finally screened six differently-expressed MeRlncRNAs and created a model for predicting the prognosis. Among the MeRlncRNAs that have been signed, it was found that some lncRNAs were up-regulated in the low-risk group, such as RP11-251M1.1, RP1-78014.1, LINC01936, and RP11-750H9.5, which were protective factors for OS. Some of them were also up-regulated in the high-risk group, such as LINC00511 and CTD-2510F5.4, which were risk factors for OS. This proves that their abnormal expression levels may be involved in the progression of cancer, including LUAD [22]. Up-expression of *LINC01936* contributed highly to the decreased risk of death, with a hazard ratio (HR) of 0.86 [23]. LINC00511, a kind of MeRlncRNA, is hugely up-regulated in colorectal carcinoma and is closely associated with the advance of malignancy [24]. In addition, Zhang et al. and Wang et al. demonstrated that LINC00511 was found to be upregulated in LUAD and enhanced LUAD malignancy [25, 26], which was consistence with our research.CTD-2510F5.4, associated with lncRNAs, is regarded as a tumor phenotype and a robust biomarker with the function of clinical diagnosis and prognosis in gastric cancer [27]. Compared to normal tissue, the expression level of RP1-78014.1 in squamous cell carcinoma and LUAD was lower, which is highly consistent with our outcomes [28]. But RP11-251M1.1 and RP11-750H9.5 are emerging novel lncRNAs, which means that our signature has both strong foreshadowing and innovative value.

In the study of LUAD patients, we observed a division based on a median risk score, effectively sorting patients into high-risk and low-risk categories. Notably, those categorized as high-risk demonstrated poorer clinical outcomes. Through rigorous analysis using multivariate Cox regression, our lncRNA signature, associated with methyltransferase activity, was identified as an independent predictor of OS. This model outperformed traditional clinical predictors in forecasting survival outcomes for LUAD, as evidenced by ROC curve analysis. To further refine our prognostic model, we developed a nomogram that accurately aligns the predicted OS with observed outcomes at one, three, and five years, showcasing its reliability and the model’s predictive precision. This level of concordance underscores the utility of our risk model, which is based on six MeRlncRNAs, as both a robust and accurate tool for future clinical research in LUAD. It opens new avenues for identifying potential biomarkers that could significantly impact the prognosis and therapeutic strategies for patients with LUAD.

GSEA enrichment assessments unearthed pathways such as the cell cycle, DNA replication, P53 signaling conduit, oncological trajectories, G2M checkpoints, cytokine receptor dialogues, and JAK-STAT communicative channels, all manifesting pronounced disparities across risk groups. The cell cycle, P53 signaling pathway, cancer pathways, DNA replication, and G2M checkpoint were all significantly greater in the high expression group, which has been linked with a poor prognosis. A transcription factor known as P53 only binds to DNA [29]. It can control the expression of certain genes and trigger apoptosis, DNA repair, and cell cycle arrest [30].

The suppression of antitumor immunity in tumors can be caused by increased cell cycle activity, which has important implications for immunotherapy [31]. DNA replication ensures that a cell’s genetic material is appropriately copied and passed on to its progeny cells [32]. However, DNA replication is susceptible to interference and damage under a variety of physiological circumstances, which can cause it to stop, impair the integrity of the genome, and even cause apoptosis, necrosis, and cancer. The fundamental cell cycle step known as the G2M checkpoint can ensure that cells won’t enter mitosis until damaged or incompletely duplicated DNA has been completely repaired. It is reported that gene expression involved in the checkpoint pathway is related to the survival results of lung cancer [33]. This study additionally discovered that immune-related signal pathways were enriched in the low-risk group, revealing that the immune cell infiltration in the immunology microenvironment has a direct relation to the prognosis of LUAD [34].

This study delved into the relationship between immune cell infiltration and the newly established risk score, conducting a comparative analysis of immune system infiltration in both high-risk and low-risk groups. The comparative findings revealed a greater prevalence of immune cells in the low-risk group, suggesting that suppression of the immune microenvironment in the high-risk group might contribute to poorer prognoses [35]. Pearson correlation analysis indicated a negative association between the risk score and various immune cells, including CD8 T cells, cytotoxic cells, dendritic cells (DC), eosinophils, immature DC (iDC), macrophages, mast cells, plasmacytoid DC (pDC), T cells, T follicular helper (TFH), and gamma delta T (Tgd) cells. Conversely, a positive correlation was observed with Th2 cells, known for their immunomodulatory impact on tumor progression. Th2 cells can facilitate tumor cell necrosis by promoting the release of type 2 cytokines within the tumor microenvironment (TME) [36].

The TME, a complex milieu of immune-suppressive and immune-activating cells, varies in the degree of tumor invasiveness across different cancer types or tumor models. Extensive research has underscored the biological relevance of lncRNAs in regulating immunity and the infiltration of immune cells within the non-small cell lung cancer (NSCLC) setting [37]. The progression, metastasis, and onset of LUAD are intimately linked with genetic discrepancies and immune function impairments within the TME [16]. Considering the pivotal role of the immune system in cancer development [17], various immunotherapeutic approaches have been devised to eradicate tumor cells [38], highlighting the importance of understanding immune cell dynamics and their association with risk scores in LUAD.

Lastly, through bioinformatics analysis, we created a ceRNA network tailored to LUAD and chose the hub lncRNA for LUAD. To our knowledge, very few research have examined lncRNAs derived from substantial sample sets. We offer a technique for locating possible lncRNA biomarkers. Additionally, we identified the LUAD ceRNA network, which will help us better comprehend the etiology of this disease.

The elucidation of MeRlncRNAs in LUAD marks a pivotal advancement in oncology, with profound implications for enhancing prognostication and refining therapeutic strategies for LUAD patients. Our discovery of a novel MeRlncRNA signature stands to revolutionize prognostic models by integrating biomarkers reflective of the disease’s molecular underpinnings, thereby improving survival prediction accuracy and deepening our understanding of tumor biology. This facilitates the identification of high-risk patients, enabling more personalized management approaches. By stratifying patients into distinct risk categories, healthcare providers can tailor follow-up and treatment strategies more effectively, optimizing patient outcomes through either intensified interventions for high-risk individuals or reduced treatment for those at lower risk, thus minimizing side effects. Additionally, the association of specific MeRlncRNAs with immune infiltration and the tumor microenvironment opens new therapeutic avenues, potentially enhancing immunotherapy efficacy and offering hope to those unresponsive to conventional treatments. The insights into MeRlncRNA functions could lead to novel therapeutic agents targeting critical pathways in LUAD pathogenesis, offering more specific and less toxic treatment alternatives. Ultimately, our research propels the field towards personalized medicine, promising LUAD patients more precise prognoses and customized treatments that significantly improve survival rates and quality of life, encapsulating a significant stride towards tailored healthcare in oncology.

Notably, the constraints posed by our sample size and potential biases merit attention, as they could influence the generalizability and interpretation of our findings. Despite rigorous methodological approaches, the representation of our sample might limit the extrapolation of our results to broader populations. Additionally, inherent biases, such as selection and measurement bias, could have impacted our analysis. Acknowledging these limitations, we propose that future research endeavors should aim to include larger and more diverse cohorts to enhance the robustness and applicability of findings. Furthermore, implementing advanced statistical techniques to adjust for potential confounders and biases could offer more nuanced insights. Finally, the mechanistic underpinnings of the MeRlncRNA signature’s influence on tumor biology and the immune microenvironment in LUAD should be further elucidated through in-depth molecular and cellular studies.

## CONCLUSION

The prognostic gene signature of LUAD associated with MeRlncRNAs that we provided, in conclusion, may offer us a comprehensive picture of the prognosis prediction for LUAD patients.

## Supplementary Materials

Supplementary Table 1

Supplementary Table 2

Supplementary Table 3

Supplementary Tables 4, 5 and 7

Supplementary Table 6

## References

[1] Jones GS, Baldwin DR. Recent advances in the management of lung cancer. Clin Med (Lond). 2018; 18:s41–6. 10.7861/clinmedicine.18-2-s41 29700092 PMC6334032

[2] Raez LE, Cardona AF, Santos ES, Catoe H, Rolfo C, Lopes G, Barrios C, Mas LA, Vallejos C, Zatarain-Barrón ZL, Caglevic C, Arrieta O. The burden of lung cancer in Latin-America and challenges in the access to genomic profiling, immunotherapy and targeted treatments. Lung Cancer. 2018; 119:7–13. 10.1016/j.lungcan.2018.02.014 29656755

[3] Travis WD. Pathology of lung cancer. Clin Chest Med. 2011; 32:669–92. 10.1016/j.ccm.2011.08.005 22054879

[4] Chen M, Liu X, Du J, Wang XJ, Xia L. Differentiated regulation of immune-response related genes between LUAD and LUSC subtypes of lung cancers. Oncotarget. 2017; 8:133–44. 10.18632/oncotarget.13346 27863400 PMC5352059

[5] Liu H, Han L, Liu Z, Gao N. Long noncoding RNA MNX1-AS1 contributes to lung cancer progression through the miR-527/BRF2 pathway. J Cell Physiol. 2019; 234:13843–50. 10.1002/jcp.28064 30618167 PMC13483036

[6] Hua Q, Mi B, Xu F, Wen J, Zhao L, Liu J, Huang G. Hypoxia-induced lncRNA-AC020978 promotes proliferation and glycolytic metabolism of non-small cell lung cancer by regulating PKM2/HIF-1α axis. Theranostics. 2020; 10:4762–78. 10.7150/thno.43839 32308748 PMC7163453

[7] Atianand MK, Caffrey DR, Fitzgerald KA. Immunobiology of Long Noncoding RNAs. Annu Rev Immunol. 2017; 35:177–98. 10.1146/annurev-immunol-041015-055459 28125358 PMC6449690

[8] Chai RC, Wu F, Wang QX, Zhang S, Zhang KN, Liu YQ, Zhao Z, Jiang T, Wang YZ, Kang CS. m^6^A RNA methylation regulators contribute to malignant progression and have clinical prognostic impact in gliomas. Aging (Albany NY). 2019; 11:1204–25. 10.18632/aging.101829 30810537 PMC6402513

[9] Barbieri I, Kouzarides T. Role of RNA modifications in cancer. Nat Rev Cancer. 2020; 20:303–22. 10.1038/s41568-020-0253-2 32300195

[10] Bai M, Sun C. M5C-Related lncRNA Predicts Lung Adenocarcinoma and Tumor Microenvironment Remodeling: Computational Biology and Basic Science. Front Cell Dev Biol. 2022; 10:885568. 10.3389/fcell.2022.885568 35592248 PMC9110831

[11] Qian X, Yang J, Qiu Q, Li X, Jiang C, Li J, Dong L, Ying K, Lu B, Chen E, Liu P, Lu Y. LCAT3, a novel m6A-regulated long non-coding RNA, plays an oncogenic role in lung cancer via binding with FUBP1 to activate c-MYC. J Hematol Oncol. 2021; 14:112. 10.1186/s13045-021-01123-0 34274028 PMC8285886

[12] Wang C, Wang W, Han X, Du L, Li A, Huang G. Methyltransferase-like 1 regulates lung adenocarcinoma A549 cell proliferation and autophagy via the AKT/mTORC1 signaling pathway. Oncol Lett. 2021; 21:330. 10.3892/ol.2021.12591 33692862 PMC7933771

[13] Ritchie ME, Phipson B, Wu D, Hu Y, Law CW, Shi W, Smyth GK. limma powers differential expression analyses for RNA-sequencing and microarray studies. Nucleic Acids Res. 2015; 43:e47. 10.1093/nar/gkv007 25605792 PMC4402510

[14] Phipson B, Lee S, Majewski IJ, Alexander WS, Smyth GK. Robust Hyperparameter Estimation Protects Against Hypervariable Genes and Improves Power To Detect Differential Expression. Ann Appl Stat. 2016; 10:946–63. 10.1214/16-AOAS920 28367255 PMC5373812

[15] Livak KJ, Schmittgen TD. Analysis of relative gene expression data using real-time quantitative PCR and the 2(-Delta Delta C(T)) Method. Methods. 2001; 25:402–8. 10.1006/meth.2001.1262 11846609

[16] Remark R, Becker C, Gomez JE, Damotte D, Dieu-Nosjean MC, Sautès-Fridman C, Fridman WH, Powell CA, Altorki NK, Merad M, Gnjatic S. The non-small cell lung cancer immune contexture. A major determinant of tumor characteristics and patient outcome. Am J Respir Crit Care Med. 2015; 191:377–90. 10.1164/rccm.201409-1671PP 25369536 PMC5447326

[17] Vinay DS, Ryan EP, Pawelec G, Talib WH, Stagg J, Elkord E, Lichtor T, Decker WK, Whelan RL, Kumara HMC, Signori E, Honoki K, Georgakilas AG, et al. Immune evasion in cancer: Mechanistic basis and therapeutic strategies. Semin Cancer Biol. 2015; 35:S185–98. 10.1016/j.semcancer.2015.03.004 25818339

[18] Skoulidis F, Heymach JV. Co-occurring genomic alterations in non-small-cell lung cancer biology and therapy. Nat Rev Cancer. 2019; 19:495–509. 10.1038/s41568-019-0179-8 31406302 PMC7043073

[19] Nandwani A, Rathore S, Datta M. LncRNAs in cancer: Regulatory and therapeutic implications. Cancer Lett. 2021; 501:162–71. 10.1016/j.canlet.2020.11.048 33359709

[20] Qi P, Du X. The long non-coding RNAs, a new cancer diagnostic and therapeutic gold mine. Mod Pathol. 2013; 26:155–65. 10.1038/modpathol.2012.160 22996375

[21] Kaliman P. Epigenetics and meditation. Curr Opin Psychol. 2019; 28:76–80. 10.1016/j.copsyc.2018.11.010 30522005

[22] Zhou W, Liu T, Saren G, Liao L, Fang W, Zhao H. Comprehensive analysis of differentially expressed long non-coding RNAs in non-small cell lung cancer. Oncol Lett. 2019; 18:1145–56. 10.3892/ol.2019.10414 31423174 PMC6607379

[23] Hou J, Yao C. Potential Prognostic Biomarkers of Lung Adenocarcinoma Based on Bioinformatic Analysis. Biomed Res Int. 2021; 2021:8859996. 10.1155/2021/8859996 33511215 PMC7822677

[24] Sun S, Xia C, Xu Y. HIF-1α induced lncRNA LINC00511 accelerates the colorectal cancer proliferation through positive feedback loop. Biomed Pharmacother. 2020; 125:110014. 10.1016/j.biopha.2020.110014 32092829

[25] Zhang Y, Xiao P, Hu X. LINC00511 enhances LUAD malignancy by upregulating GCNT3 via miR-195-5p. BMC Cancer. 2022; 22:389. 10.1186/s12885-022-09459-7 35399076 PMC8994914

[26] Wang Y, Mei X, Song W, Wang C, Qiu X. LncRNA LINC00511 promotes COL1A1-mediated proliferation and metastasis by sponging miR-126-5p/miR-218-5p in lung adenocarcinoma. BMC Pulm Med. 2022; 22:272. 10.1186/s12890-022-02070-3 35842617 PMC9287882

[27] Wang Z, Qin B. Prognostic and clinicopathological significance of long noncoding RNA CTD-2510F5.4 in gastric cancer. Gastric Cancer. 2019; 22:692–704. 10.1007/s10120-018-00911-x 30560474 PMC6570689

[28] Chen WJ, Tang RX, He RQ, Li DY, Liang L, Zeng JH, Hu XH, Ma J, Li SK, Chen G. Clinical roles of the aberrantly expressed lncRNAs in lung squamous cell carcinoma: a study based on RNA-sequencing and microarray data mining. Oncotarget. 2017; 8:61282–304. 10.18632/oncotarget.18058 28977863 PMC5617423

[29] Sakaguchi K, Herrera JE, Saito S, Miki T, Bustin M, Vassilev A, Anderson CW, Appella E. DNA damage activates p53 through a phosphorylation-acetylation cascade. Genes Dev. 1998; 12:2831–41. 10.1101/gad.12.18.2831 9744860 PMC317174

[30] Liebl MC, Hofmann TG. The Role of p53 Signaling in Colorectal Cancer. Cancers (Basel). 2021; 13:2125. 10.3390/cancers13092125 33924934 PMC8125348

[31] Li J, Stanger BZ. Cell Cycle Regulation Meets Tumor Immunosuppression. Trends Immunol. 2020; 41:859–63. 10.1016/j.it.2020.07.010 32800703 PMC12118812

[32] Mazouzi A, Velimezi G, Loizou JI. DNA replication stress: causes, resolution and disease. Exp Cell Res. 2014; 329:85–93. 10.1016/j.yexcr.2014.09.030 25281304

[33] Eymin B, Gazzeri S. Role of cell cycle regulators in lung carcinogenesis. Cell Adh Migr. 2010; 4:114–23. 10.4161/cam.4.1.10977 20139697 PMC2852568

[34] Fan T, Zhu M, Wang L, Liu Y, Tian H, Zheng Y, Tan F, Sun N, Li C, He J. Immune profile of the tumor microenvironment and the identification of a four-gene signature for lung adenocarcinoma. Aging (Albany NY). 2020; 13:2397–417. 10.18632/aging.202269 33318300 PMC7880407

[35] Mao X, Xu J, Wang W, Liang C, Hua J, Liu J, Zhang B, Meng Q, Yu X, Shi S. Crosstalk between cancer-associated fibroblasts and immune cells in the tumor microenvironment: new findings and future perspectives. Mol Cancer. 2021; 20:131. 10.1186/s12943-021-01428-1 34635121 PMC8504100

[36] Nishimura T, Iwakabe K, Sekimoto M, Ohmi Y, Yahata T, Nakui M, Sato T, Habu S, Tashiro H, Sato M, Ohta A. Distinct role of antigen-specific T helper type 1 (Th1) and Th2 cells in tumor eradication in vivo. J Exp Med. 1999; 190:617–27. 10.1084/jem.190.5.617 10477547 PMC2195611

[37] Sun J, Zhang Z, Bao S, Yan C, Hou P, Wu N, Su J, Xu L, Zhou M. Identification of tumor immune infiltration-associated lncRNAs for improving prognosis and immunotherapy response of patients with non-small cell lung cancer. J Immunother Cancer. 2020; 8:e000110. 10.1136/jitc-2019-000110 32041817 PMC7057423

[38] Hirsch FR, Scagliotti GV, Mulshine JL, Kwon R, Curran WJ Jr, Wu YL, Paz-Ares L. Lung cancer: current therapies and new targeted treatments. Lancet. 2017; 389:299–311. 10.1016/S0140-6736(16)30958-8 27574741

