# Supplementary material for: Identification of a methyltransferase-related long noncoding RNA signature as a novel prognosis biomarker for lung adenocarcinoma
Source: Aging (Albany NY). 2024 May 20;16(10):8747–71. doi: 10.18632/aging.205837 (PMC11164517; doi:10.18632/aging.205837)
Supplement: Supplementary Tables 4, 5 and 7 [file aging-16-205837-s004.pdf]

SUPPLEMENTARY TABLES

Supplementary Table 4. The mRNA-lncRNA network between the five methyltransferase-related genes and DElncRNAs.

mRNA	lncRNA	cor	*p*-value
ENSG00000106462|protein_coding|EZH2	ENSG00000268388|lncRNA|FENDRR	−0.30632682	1.11E-10
ENSG00000162851|protein_coding|TFB2M	ENSG00000268388|lncRNA|FENDRR	−0.398287677	1.99E-19
ENSG00000182004|protein_coding|SNRPE	ENSG00000268388|lncRNA|FENDRR	−0.410341825	8.42E-21
ENSG00000278619|protein_coding|MRM1	ENSG00000268388|lncRNA|FENDRR	−0.35152291	1.31E-14
ENSG00000162851|protein_coding|TFB2M	ENSG00000238018|lncRNA|AC093110.3	−0.439446064	2.29E-24
ENSG00000182004|protein_coding|SNRPE	ENSG00000238018|lncRNA|AC093110.3	−0.454915104	2.09E-26
ENSG00000278619|protein_coding|MRM1	ENSG00000238018|lncRNA|AC093110.3	−0.342945197	8.16E-14
ENSG00000106462|protein_coding|EZH2	ENSG00000228401|lncRNA|RP11-251M1.1	−0.301387692	2.72E-10
ENSG00000162851|protein_coding|TFB2M	ENSG00000228401|lncRNA|RP11-251M1.1	−0.347354355	3.21E-14
ENSG00000182004|protein_coding|SNRPE	ENSG00000228401|lncRNA|RP11-251M1.1	−0.339178511	1.78E-13
ENSG00000278619|protein_coding|MRM1	ENSG00000228401|lncRNA|RP11-251M1.1	−0.318974385	1.02E-11
ENSG00000106462|protein_coding|EZH2	ENSG00000257894|lncRNA|RP1-78O14.1	−0.329857913	1.20E-12
ENSG00000182004|protein_coding|SNRPE	ENSG00000257894|lncRNA|RP1-78O14.1	−0.326869158	2.19E-12
ENSG00000278619|protein_coding|MRM1	ENSG00000257894|lncRNA|RP1-78O14.1	−0.305690169	1.25E-10
ENSG00000106462|protein_coding|EZH2	ENSG00000267107|lncRNA|PCAT19	−0.31904007	1.01E-11
ENSG00000162851|protein_coding|TFB2M	ENSG00000267107|lncRNA|PCAT19	−0.370937651	1.63E-16
ENSG00000182004|protein_coding|SNRPE	ENSG00000267107|lncRNA|PCAT19	−0.363586645	8.96E-16
ENSG00000278619|protein_coding|MRM1	ENSG00000267107|lncRNA|PCAT19	−0.345486563	4.78E-14
ENSG00000106462|protein_coding|EZH2	ENSG00000243961|lncRNA|PARAL1	−0.317282155	1.42E-11
ENSG00000162851|protein_coding|TFB2M	ENSG00000243961|lncRNA|PARAL1	−0.303503227	1.86E-10
ENSG00000182004|protein_coding|SNRPE	ENSG00000243961|lncRNA|PARAL1	−0.311264988	4.42E-11
ENSG00000278619|protein_coding|MRM1	ENSG00000243961|lncRNA|PARAL1	−0.32632611	2.43E-12
ENSG00000106462|protein_coding|EZH2	ENSG00000224397|lncRNA|PELATON	−0.311576011	4.18E-11
ENSG00000162851|protein_coding|TFB2M	ENSG00000224397|lncRNA|PELATON	−0.399089074	1.62E-19
ENSG00000182004|protein_coding|SNRPE	ENSG00000224397|lncRNA|PELATON	−0.413106073	4.00E-21
ENSG00000278619|protein_coding|MRM1	ENSG00000224397|lncRNA|PELATON	−0.356601881	4.29E-15
ENSG00000162851|protein_coding|TFB2M	ENSG00000276850|lncRNA|CH17-360D5.2	−0.315518346	1.99E-11
ENSG00000278619|protein_coding|MRM1	ENSG00000276850|lncRNA|CH17-360D5.2	−0.303817355	1.76E-10
ENSG00000106462|protein_coding|EZH2	ENSG00000235997|lncRNA|LINC01936	−0.342983154	8.11E-14
ENSG00000162851|protein_coding|TFB2M	ENSG00000235997|lncRNA|LINC01936	−0.371864993	1.32E-16
ENSG00000182004|protein_coding|SNRPE	ENSG00000235997|lncRNA|LINC01936	−0.403529729	5.13E-20
ENSG00000278619|protein_coding|MRM1	ENSG00000235997|lncRNA|LINC01936	−0.372102438	1.25E-16
ENSG00000106462|protein_coding|EZH2	ENSG00000261269|lncRNA|RP11-389C8.2	−0.347474072	3.14E-14
ENSG00000162851|protein_coding|TFB2M	ENSG00000261269|lncRNA|RP11-389C8.2	−0.44316373	7.57E-25
ENSG00000182004|protein_coding|SNRPE	ENSG00000261269|lncRNA|RP11-389C8.2	−0.469694218	1.86E-28
ENSG00000278619|protein_coding|MRM1	ENSG00000261269|lncRNA|RP11-389C8.2	−0.347832017	2.91E-14
ENSG00000162851|protein_coding|TFB2M	ENSG00000231993|lncRNA|EP300-AS1	−0.333237742	6.08E-13
ENSG00000182004|protein_coding|SNRPE	ENSG00000231993|lncRNA|EP300-AS1	−0.391796678	1.03E-18
ENSG00000106462|protein_coding|EZH2	ENSG00000234456|lncRNA|MAGI2-AS3	−0.346987097	3.47E-14
ENSG00000162851|protein_coding|TFB2M	ENSG00000234456|lncRNA|MAGI2-AS3	−0.356327825	4.55E-15
ENSG00000182004|protein_coding|SNRPE	ENSG00000234456|lncRNA|MAGI2-AS3	−0.435490975	7.31E-24
ENSG00000278619|protein_coding|MRM1	ENSG00000234456|lncRNA|MAGI2-AS3	−0.374763748	6.66E-17
ENSG00000162851|protein_coding|TFB2M	ENSG00000267280|lncRNA|TBX2-AS1	−0.326559974	2.32E-12

ENSG00000106462|protein_coding|EZH2	ENSG00000214708|lncRNA|AC090616.2	−0.362249996	1.21E-15
ENSG00000106462|protein_coding|EZH2	ENSG00000186594|lncRNA|MIR22HG	−0.341161534	1.18E-13
ENSG00000162851|protein_coding|TFB2M	ENSG00000186594|lncRNA|MIR22HG	−0.303040006	2.02E-10
ENSG00000182004|protein_coding|SNRPE	ENSG00000186594|lncRNA|MIR22HG	−0.376061831	4.90E-17
ENSG00000278619|protein_coding|MRM1	ENSG00000186594|lncRNA|MIR22HG	−0.344779073	5.54E-14
ENSG00000106462|protein_coding|EZH2	ENSG00000255399|lncRNA|TBX5-AS1	−0.406271365	2.49E-20
ENSG00000162851|protein_coding|TFB2M	ENSG00000255399|lncRNA|TBX5-AS1	−0.395630987	3.92E-19
ENSG00000182004|protein_coding|SNRPE	ENSG00000255399|lncRNA|TBX5-AS1	−0.487013718	5.43E-31
ENSG00000278619|protein_coding|MRM1	ENSG00000255399|lncRNA|TBX5-AS1	−0.391735627	1.05E-18
ENSG00000182004|protein_coding|SNRPE	ENSG00000228288|lncRNA|PCAT6	0.336298437	3.24E-13
ENSG00000278619|protein_coding|MRM1	ENSG00000228288|lncRNA|PCAT6	0.390509979	1.42E-18
ENSG00000106462|protein_coding|EZH2	ENSG00000227036|lncRNA|LINC00511	0.361383405	1.47E-15
ENSG00000106462|protein_coding|EZH2	ENSG00000255197|lncRNA|RP11-750H9.5	−0.302422331	2.25E-10
ENSG00000162851|protein_coding|TFB2M	ENSG00000255197|lncRNA|RP11-750H9.5	−0.453505748	3.23E-26
ENSG00000182004|protein_coding|SNRPE	ENSG00000255197|lncRNA|RP11-750H9.5	−0.531668814	3.08E-38
ENSG00000278619|protein_coding|MRM1	ENSG00000255197|lncRNA|RP11-750H9.5	−0.384650018	6.10E-18
ENSG00000278619|protein_coding|MRM1	ENSG00000249859|lncRNA|PVT1	0.349424575	2.06E-14
ENSG00000162851|protein_coding|TFB2M	ENSG00000273837|lncRNA|LLNLR-470E3.1	−0.393563246	6.63E-19
ENSG00000182004|protein_coding|SNRPE	ENSG00000273837|lncRNA|LLNLR-470E3.1	−0.339760758	1.58E-13
ENSG00000278619|protein_coding|MRM1	ENSG00000273837|lncRNA|LLNLR-470E3.1	−0.328104055	1.71E-12
ENSG00000106462|protein_coding|EZH2	ENSG00000255717|lncRNA|SNHG1	0.578794396	3.72E-47
ENSG00000182004|protein_coding|SNRPE	ENSG00000255717|lncRNA|SNHG1	0.398378545	1.95E-19
ENSG00000278619|protein_coding|MRM1	ENSG00000255717|lncRNA|SNHG1	0.360887414	1.64E-15
ENSG00000106462|protein_coding|EZH2	ENSG00000225383|lncRNA|SFTA1P	−0.401031022	9.83E-20
ENSG00000106462|protein_coding|EZH2	ENSG00000280206|lncRNA|CTB-193M12.5	0.354833938	6.33E-15
ENSG00000106462|protein_coding|EZH2	ENSG00000261373|lncRNA|VPS9D1-AS1	0.314591354	2.37E-11
ENSG00000278619|protein_coding|MRM1	ENSG00000261373|lncRNA|VPS9D1-AS1	0.362595128	1.12E-15
ENSG00000278619|protein_coding|MRM1	ENSG00000243479|lncRNA|MNX1-AS1	0.322368782	5.30E-12
ENSG00000106462|protein_coding|EZH2	ENSG00000265415|lncRNA|CTD-2510F5.4	0.612540563	1.72E-54
ENSG00000182004|protein_coding|SNRPE	ENSG00000265415|lncRNA|CTD-2510F5.4	0.331806503	8.12E-13
ENSG00000278619|protein_coding|MRM1	ENSG00000242125|lncRNA|SNHG3	0.341799928	1.03E-13
ENSG00000106462|protein_coding|EZH2	ENSG00000267751|lncRNA|BSG-AS1	0.320670483	7.39E-12
ENSG00000182004|protein_coding|SNRPE	ENSG00000267751|lncRNA|BSG-AS1	0.351302207	1.37E-14
ENSG00000162851|protein_coding|TFB2M	ENSG00000234614|lncRNA|C2CD4D-AS1	0.341408017	1.12E-13
ENSG00000106462|protein_coding|EZH2	ENSG00000232677|lncRNA|LINC00665	0.34197833	9.99E-14
ENSG00000182004|protein_coding|SNRPE	ENSG00000232677|lncRNA|LINC00665	0.31332984	3.00E-11
ENSG00000278619|protein_coding|MRM1	ENSG00000249007|lncRNA|RP11-510N19.5	0.325740707	2.72E-12

Supplementary Table 5. The regression coefficient.

Gene	Coef
ENSG00000228401|lncRNA|RP11-251M1.1	−0.081695395
ENSG00000257894|lncRNA|RP1-78O14.1	−0.022561732
ENSG00000235997|lncRNA|LINC01936	−0.071522442
ENSG00000227036|lncRNA|LINC00511	0.012186292
ENSG00000255197|lncRNA|RP11-750H9.5	−0.10909988
ENSG00000265415|lncRNA|CTD-2510F5.4	0.105977347

Supplementary Table 7. The lncRNA-miRNA-mRNA regulatory network.

miRNA	lncRNA	mRNA
hsa-let-7a-5p	RP11-251M1.1	TAF4
hsa-let-7a-5p	RP11-251M1.1	TAF4
hsa-let-7a-5p	LINC01936	TAF4
hsa-let-7a-5p	LINC01936	TAF4
hsa-let-7a-5p	LINC00511	TAF4
hsa-let-7a-5p	LINC00511	TAF4
hsa-let-7a-5p	CTD-2510F5.4	TAF4
hsa-let-7a-5p	CTD-2510F5.4	TAF4
hsa-let-7d-5p	LINC01936	PRMT1
hsa-let-7d-5p	RP11-750H9.5	PRMT1
hsa-let-7d-5p	RP11-251M1.1	PRMT1
hsa-let-7d-5p	CTD-2510F5.4	PRMT1
hsa-let-7d-5p	LINC00511	PRMT1
hsa-miR-103a-3p	LINC01936	PRPF31
hsa-miR-103a-3p	LINC00511	PRPF31
hsa-miR-103a-3p	RP11-251M1.1	PRPF31
hsa-miR-146a-5p	RP11-251M1.1	RBBP7
hsa-miR-146a-5p	LINC00511	RBBP7
hsa-miR-155-5p	LINC00511	ERH
hsa-miR-155-5p	LINC00511	CXXC1
hsa-miR-21-5p	LINC00511	MRM3
hsa-miR-21-5p	LINC00511	MCRS1
hsa-miR-302a-3p	LINC00511	JARID2
hsa-miR-302a-3p	RP11-251M1.1	JARID2
hsa-miR-302a-3p	LINC01936	JARID2
hsa-miR-302a-5p	LINC00511	METTL8
hsa-miR-302a-5p	RP11-251M1.1	METTL8
hsa-miR-302a-5p	LINC01936	METTL8
hsa-miR-302b-3p	RP11-251M1.1	METTL3
hsa-miR-302b-3p	LINC00511	METTL3
hsa-miR-3198	LINC01936	CBX5
hsa-miR-3198	LINC01936	CBX5
hsa-miR-3198	LINC00511	CBX5
hsa-miR-3198	LINC00511	CBX5
hsa-miR-3198	RP1-78O14.1	CBX5
hsa-miR-3198	RP1-78O14.1	CBX5
hsa-miR-3198	RP11-251M1.1	CBX5
hsa-miR-3198	RP11-251M1.1	CBX5
hsa-miR-3654	LINC00511	CLNS1A
hsa-miR-4426	LINC01936	WDR77
hsa-miR-4426	LINC01936	RBBP5
hsa-miR-4426	RP11-251M1.1	WDR77
hsa-miR-4426	RP11-251M1.1	RBBP5
hsa-miR-4426	LINC00511	WDR77
hsa-miR-4426	LINC00511	RBBP5
hsa-miR-4449	RP11-750H9.5	FTSJ1

hsa-miR-4449	LINC00511	FTSJ1
hsa-miR-4449	RP11-251M1.1	FTSJ1
hsa-miR-4709-3p	LINC01936	RBM15B
hsa-miR-4709-3p	RP1-78O14.1	RBM15B
hsa-miR-4709-3p	RP11-251M1.1	RBM15B
hsa-miR-4709-3p	LINC00511	RBM15B
hsa-miR-4724-3p	LINC00511	CMTR1
hsa-miR-492	LINC00511	TRMT44
hsa-miR-492	CTD-2510F5.4	TRMT44
hsa-miR-492	LINC01936	TRMT44
hsa-miR-492	RP11-251M1.1	TRMT44
hsa-miR-6747-3p	LINC00511	DPY30
hsa-miR-6747-3p	RP11-251M1.1	DPY30
hsa-miR-6747-3p	RP1-78O14.1	DPY30
hsa-miR-6747-3p	LINC01936	DPY30
hsa-miR-6798-3p	RP11-251M1.1	RUVBL2
hsa-miR-6798-3p	RP11-251M1.1	RUVBL2
hsa-miR-6798-3p	LINC01936	RUVBL2
hsa-miR-6798-3p	LINC01936	RUVBL2
hsa-miR-6798-3p	LINC00511	RUVBL2
hsa-miR-6798-3p	LINC00511	RUVBL2
hsa-miR-92a-3p	RP11-251M1.1	RBBP5
hsa-miR-92a-3p	LINC00511	RBBP5
hsa-miR-92a-3p	LINC01936	RBBP5
